# Overexpression of the Kiwifruit Transcription Factor *AaMYB44* Decreases the Cold Tolerance in *Arabidopsis thaliana*

**DOI:** 10.3390/plants13223126

**Published:** 2024-11-06

**Authors:** Yihang Li, Miaomiao Lin, Qina Zhang, Peng Zhang, Zhenzhen Zhang, Yukuo Li, Leiming Sun, Sumei Li, Congcong Li, Dixin Chen, Xiujuan Qi

**Affiliations:** 1 College of Horticulture and Plant Protection, Henan University of Science and Technology, Luoyang 471000, China; yihangli412@126.com; 2 National Key Laboratory for Germplasm Innovation & Utilization of Horticultural Crops, Zhengzhou Fruit Research Institute, Chinese Academy of Agricultural Sciences, Zhengzhou 450009, China; linmiaomiao@caas.cn (M.L.); zhang_qina99@163.com (Q.Z.); 13683927915@163.com (P.Z.); 18703882835@163.com (Z.Z.); liyukuo@caas.cn (Y.L.); sunleiming@caas.cn (L.S.); 15615642046@163.com (S.L.); 18705887542@163.com (C.L.); 3 Zhongyuan Research Center, Chinese Academy of Agricultural Sciences, Xinxiang 453500, China

**Keywords:** kiwifruit, *AaMYB44*, transcriptome, mechanism of freezing tolerance

## Abstract

Cold stress is one of the main abiotic stresses that affect the development and growth of kiwifruit (*Actinidia arguta*). Herein, we analyzed the transcriptomic data of *A. arguta* dormant shoots in response to low-temperature treatment, identified 52 MYB genes, and constructed a phylogenetic tree based on the encoded protein sequences. Then, the effect of one *MYB* gene on cold tolerance was analyzed. This gene had an open reading frame of 837 bp long and encoded 279 amino acids. Sequence alignment and phylogenetic analysis revealed that this gene belongs to the *R2R3-MYB* family and was named *AaMYB44* based on its homology to other *MYB* family members. Quantitative real-time PCR revealed that *AaMYB44* expression was significantly induced by low temperatures but exhibited the opposite trend in cold-tolerant genotypes. Subcellular localization assays revealed the nuclear localization of the AaMYB44 protein. Furthermore, *AaMYB44* was transformed into *Arabidopsis thaliana* (*A. thaliana*) via inflorescence infection, and physiological and biochemical tests revealed that the cold resistance and antioxidant capacity of the transgenic *A. thaliana* were lower than those of wild-type plants. Overall, *AaMYB44* might play a negative regulatory role in response to cold stress, providing new insight into the mechanism of cold tolerance.

## 1. Introduction

Cold stress is an important abiotic stress that directly affects the growth and development of plants, including the kiwifruit (*Actinidia arguta*) [1]. Kiwifruits are widely distributed throughout China, from N20° to N50°; however, commercially cultivated kiwifruits are grown mainly south of the Yellow River in China and have poor tolerance to freezing conditions. In recent years, due to the occurrence of extremely cold weather, freezing damage has severely affected the kiwifruit industry [2]. For example, in different years, the United States, Italy, and Iran reported kiwifruit frost damage [3,4,5].

To survive cold stress, plants have developed the ability to resist low temperatures, and transcriptional regulation is an important means by which plants mediate this resistance [6]. Transcription factors (TFs) are key factors that control gene expression in organisms. Under different environmental conditions, TFs with specific transcription regulatory regions (promoters, enhancers, etc.) regulate target gene expression and play an extremely important role in increasing stress resistance via the plant defense system [7]. The MYB TF family is one of the largest TF families in plants [8], and many studies have indicated that MYB TFs are involved in the development, secondary metabolism, hormone signaling, disease resistance, and tolerance of plants to biological and abiotic stresses [9,10,11]. The *R2R3-MYB* subfamily contains two conserved R2 and R3 repeats in the MYB-binding domain, as well as a regulatory domain for activation or inhibition in the C-terminal variable region. *R2R3-MYB* subfamily members have many functions, including cell differentiation, secondary metabolism, environmental stress, and the invasion of pests and diseases. *OsMYB2* encodes a stress-responsive MYB TF that plays a regulatory role in the tolerance of rice to salt, cold, and dehydration stress [12]. In pineapples, *AcoMYB1* expression is induced after low-temperature treatment, and this gene positively regulates the response to cold stress [13]. The apple’s E3 ubiquitin ligase gene, *MdMIEL1,* interacts with *MdMYB308L* to promote the ubiquitination and degradation of the MdMYB308L protein, thereby reducing the tolerance of these plants to cold stress [14]. MYB TFs also negatively regulate the response of plants to abiotic stress. *AhMYB44-16* negatively regulates plant drought tolerance through its involvement in ABA-dependent stress response pathways [15]. *Arabidopsis thaliana AtMYB15*-overexpressing plants exhibit reduced tolerance to low-temperature stress [16]. Moreover, *FtMYB10* plays a key role in the regulation of ABA feedback signaling and is a novel negative regulator of salt and drought stress tolerance in plants [17].

*AdMYB7* is an activator of the *AdLCY-β* gene promoter and is, therefore, a key component of the kiwifruit carotenoid pathway [18]. The candidate gene *MYB110*, which is associated with fruit color, was found on chromosome 9 of kiwifruit via quantitative trait locus (QTL) mapping; this gene has been shown to regulate fruit color [19]. In total, 181 AcMYB TFs were identified from the kiwifruit genome and were found to be unevenly distributed across 29 chromosomes [2]. The interaction between *AcMYB10* and *AcbHLH42* in kiwifruit strongly activates the biosynthesis of anthocyanin by enhancing the transcription of *AcLDOX* and *AcF3GT* [20]. However, how the *MYB* gene family is involved in the resistance of kiwifruit to cold stress is unknown.

*A. arguta* is widely distributed in China. Studies have indicated that this species strongly resists cold stress and has great commercial cultivation value [21,22,23]. In this study, the transcriptomic data of the dormant shoots from previous studies on kiwifruit in response to low-temperature treatment were used to identify the *MYB* gene families [23]. The full-length *MYB* gene was subsequently cloned and named *AaMYB44* based on its homology to other *MYB* family members. The AaMYB44 protein is subcellularly localized in the nucleus. The overexpression of *AaMYB44* in *A. thaliana* results in a decreased resistance to cold stress, which implies that this gene plays a negative role in cold tolerance regulation. Overall, this study provides genetic resources and a theoretical basis for the molecularly directed cultivation of cold-resistant kiwifruit.

## 2. Results

### 2.1. Identification and Phylogeny of AaMYB Genes

**Figure 1:**
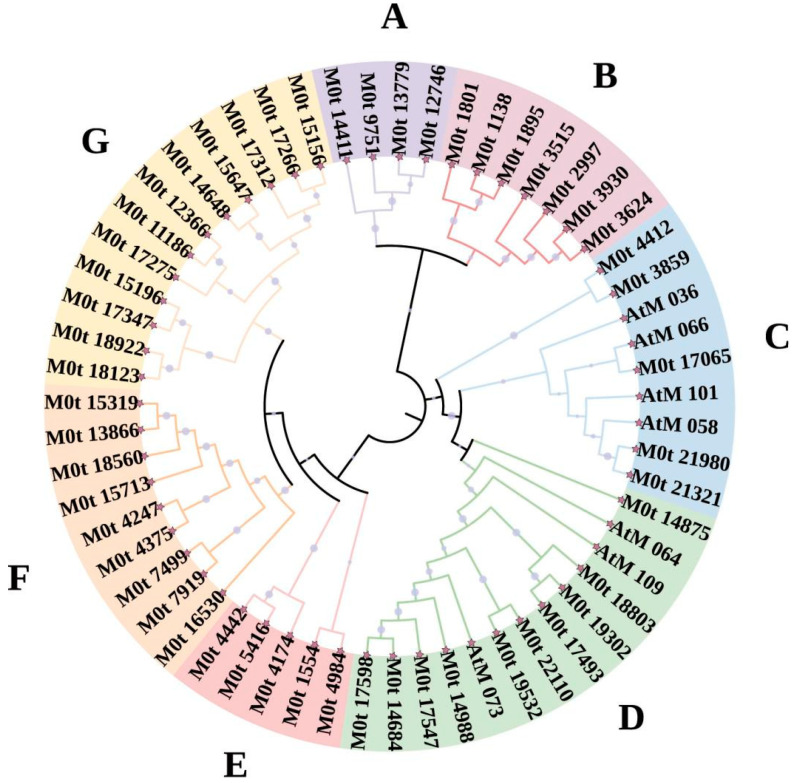
The phylogenetic tree of the *A. arguta MYB* gene family. A, B, C, D, E, F, and G indicate Group A, Group B, Group C, Group D, Group E, Group F, and Group G, respectively.

**Figure 2:**
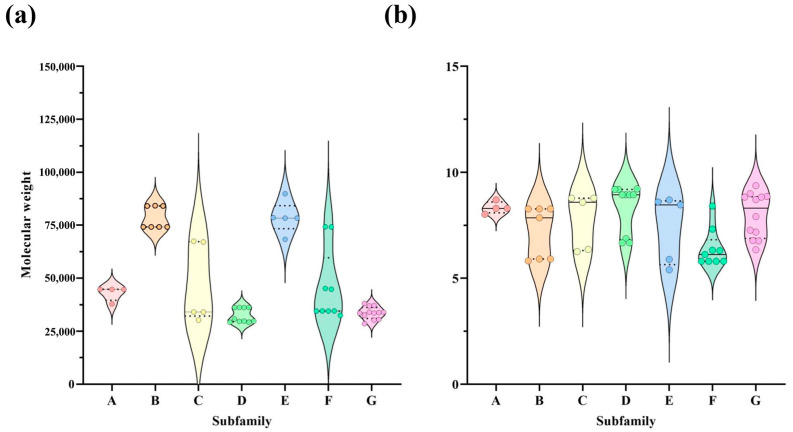
The chemical characteristics of the *AaMYB* gene family in kiwifruit. (**a**) The molecular weights of the members in the *AaMYB* family. (**b**) The protein isoelectric points in the *AaMYB* family. A, B, C, D, E, F, and G indicate Group A, Group B, Group C, Group D, Group E, Group F, and Group G, respectively.

A total of 52 *AaMYB* genes were identified through the analysis of previous transcriptomic data from *A. arguta* dormant shoots (Supplementary Table S1), and seven *A. thaliana MYB* genes were obtained from NCBI (Supplementary Table S2). The structural domains of these genes were analyzed using InterPro (https://www.ebi.ac.uk/interpro, accessed data: 21 April 2024) [23]. According to the branches of the phylogenetic tree, the *AaMYB* members were divided into seven subfamilies, with four, seven, five, ten, five, nine, and twelve members in subfamily Groups A to G, respectively (Supplementary Table S3) (Figure 1). All of the *A. thaliana MYB* genes were in Groups C and D. To further characterize the *AaMYB* genes, we subsequently analyzed their isoelectric points and molecular weights (Supplementary Table S4). The genes in Groups A, C, D, F, and G had similar molecular weights, primarily in the range of 30,000 Da to 40,000 Da, while the molecular weights of the genes in Groups B and E were concentrated mainly at 75,000 Da (Figure 2a). The isoelectric points of the genes in all groups were consistent, ranging mainly from five to ten but mostly concentrated between eight and nine. Moreover, the isoelectric points of the genes in Group F were approximately six and seven (Figure 2b). Finally, BLAST search identified 52 homologous *MYB* genes in the kiwifruit Hongyang V2 genome (https://kiwifruitgenome.org/organism/2 accessed data: 21 April 2024) [24] (Supplementary Table S1).

### 2.2. The Conserved Domains and Motifs of the AaMYBs

**Figure 3:**
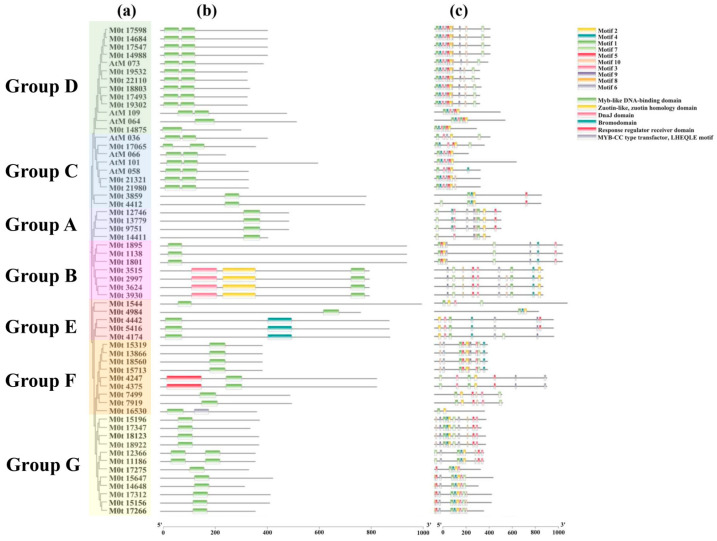
The domain and motif analysis of the AaMYB proteins in *A. arguta*. (**a**) The phylogenetic tree of the *AaMYB* gene family. (**b**) the domain analysis of the AaMYB proteins. (**c**) The motif analysis of the AaMYB proteins.

An analysis of the domains and motif distribution can be used to understand the structural characteristics of the proteins encoded by the *AaMYB* genes [24,25]. This analysis indicated that the proteins encoded by the most *AaMYB* genes in Group C and Group D had two MYB-like DNA-binding domains (Figure 3a,b), as did M0t12366 and M0t11186 in Group G, suggesting that these *MYB* genes were typical *R2R3-MYB* genes (Figure 3b). Motif analysis revealed the presence of motif 1 and motif 2 in all proteins encoded by these *MYB* genes. Motif 8 was present in Groups B, D, F and G only, whereas motif 9 was not present in Group C. M0t16530 in Group F, M0t17275 in Group G, M0t1554 and M0t4984 in Group E, and M0t3859 and M0t4412 in Group C had only less than five or equal motifs. Overall, these results suggest that the genes in different *AaMYB* subfamilies have different functions.

### 2.3. The Expression of AaMYBs in the Two Genotypes of A. arguta and the Prediction of the AcMYB44 Regulatory Network

**Figure 4:**
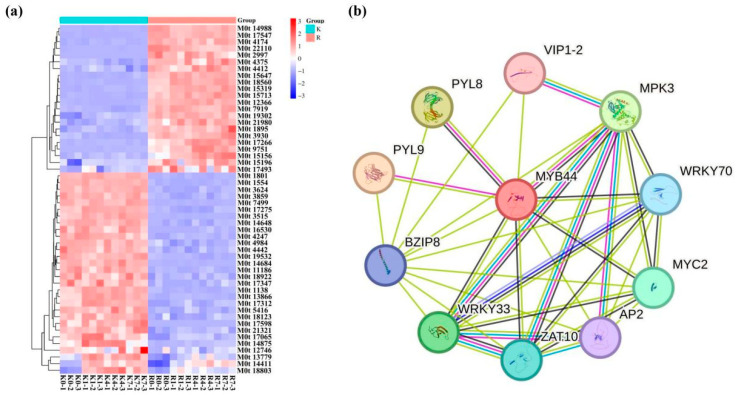
The expression patterns of genes in the *MYB* family in two genotypes of *A. arguta* exposed to low-temperature stress and prediction of the MYB44-interacting proteins in *A. thaliana.* (**a**) A heatmap of *MYB* genes exposed to low-temperature stress. K indicates KL plants (cold-tolerant genotype); R indicates RB-3 plants (cold-sensitive genotype); K0, K1, K4, and K7 indicate the dormant shoots of KL plants treated at −25 °C for 0 h, 1 h, 4 h, and 7 h, respectively; and R0, R1 R4, and R7 indicate the dormant shoots of RB-3 plants treated at −25 °C for 0 h, 1 h, 4 h, and 7 h, respectively. (**b**) The protein regulatory networks of AaMYB44. Blue, purple: the known interactions from curated databases and experimentally determined interactions, respectively; green, pink: the predicted interactions, gene neighborhood, gene fusions, and gene co-occurrence; yellow-green, black, and blue: others, including text mining, co-expression, and protein homology, respectively.

The expression levels of the *AaMYB* family members and the two genotypes of *A. arguta* with different extents of cold tolerance were used to create a cluster heatmap (Supplementary Table S5). Thirty *AaMYB* genes presented high levels of expression in KL plants (cold-tolerant genotype) in contrast to RB-3 plants (cold-sensitive genotype); moreover, the remaining 20 *AaMYB* genes presented the opposite expression pattern (Figure 4a). The expression of M0t13779, M0t14411, and M0t18803 increased significantly as the duration of cold treatment increased; among them, M0t18803, an *R2R3-MYB* member that belongs to Group D, displayed the most prominent increase. The STRING database was used to predict the regulatory network of AaMYB44 TFs in *A. arguta*. AaMYB44 interacted with MYC2, PYL9, WRKY33, BZIP8, VIP1-2, WRKY70, PYL8, ZAT10, MPK3, and AP2 (Figure 4b). Specifically, there was a co-expression prediction between AaMYB44 and PYL8, MPK3, WRKY70, MYC2, ZAT10, and WRKY33.

### 2.4. Cloning and Sequence Analysis of AaMYB44

**Figure 5:**
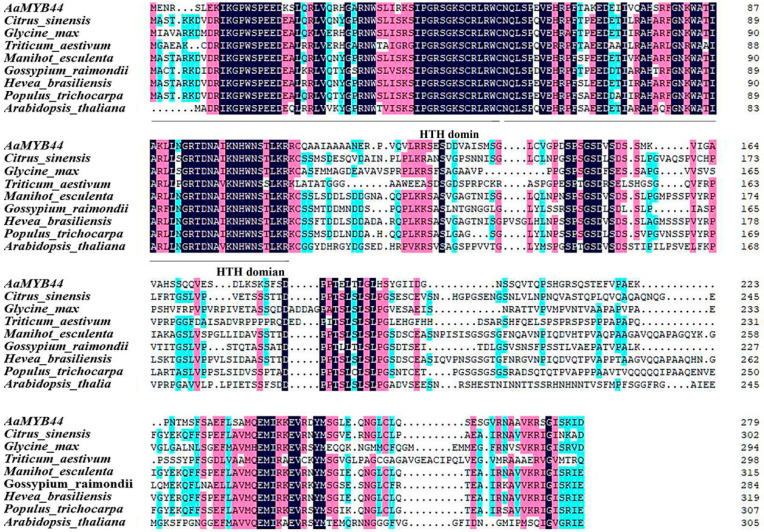
Multiple sequence alignment of homologous MYB proteins. Dark blue highlights identical residues, while similar residues are marked in pink or light blue, and black lines represent two HTH domains. *Citrus sinensis* (KAH9758124.1); *Glycine max* (XP 003524661.1); *Triticum aestivum* (XP 044402740.1); *Manihot esculenta* (XP 021601648.1); *Gossypium raimondii* (XP 012446627.1); *Hevea brasiliensis* (XP 021678755.2); *Populus trichocarpa* (XP 024451715.2); and *Arabidopsis thaliana* (CAA90809.1).

The *R2R3-MYB* subfamily is involved in plant growth and development, secondary metabolism, biotic stress, and abiotic stress [26]. We selected R2R3-MYB and M0t18803 as candidate genes because they respond to low temperatures in different cold-tolerant genotypes. The full-length M0t18803 gene was isolated and identified and included an 840 bp coding sequence (CDS), which encoded 280 amino acids (aa). This gene was found to be homologous to *A. thaliana AtMYB44*; thus, it was named *AaMYB44* (Figure 5). The tool ProtParam (http://web.expasy.org/protparam/, accessed on 21 April 2024) was used to analyze the primary structure of the *AaMYB44* protein. The main aa present were tryptophan (14.3%), arginine (7.2%), alanine (6.8%), glutamic acid (6.8%), glycine (6.8%), leucine (6.8%), and lysine (6.5%). AaMYB44 has a relative molecular mass of 30,719.56, a molecular formula of C_1316_H_2131_N_403_O_421_S_12_, an isoelectric point of 9.22, and an instability coefficient of 59.73 (>40). The predicted protein structure of AaMYB44 (https://swissmodel.ExPASy.org/, accessed on 21 April 2024) contained a helix–turn–helix (HTH) motif characteristic of the *R2R3-MYB* subfamily. The sequence of *AaMYB44* was submitted to the National Center for Biotechnology Information (GenBank no: PP998223).

**Figure 6:**
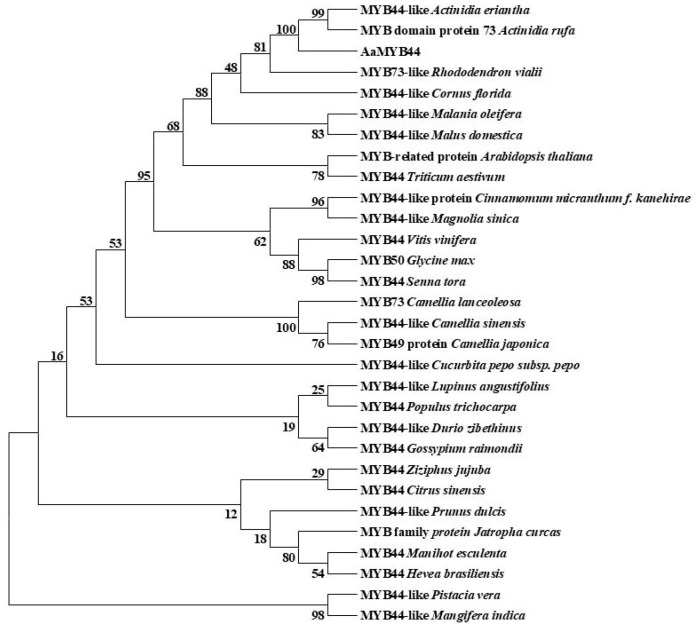
Phylogenetic tree analysis of *AaMYB44.* Numbers above/below branches represented bootstrap values (from 1000 replicates).

Alignment of the AaMYB44 sequence with the MYB protein sequences in other species revealed its greater similarity to the MYB protein sequences in other species (Supplementary Table S6). Moreover, these sequences had the same conserved HTH motif (Figure 5). A phylogenetic tree analysis revealed that the AaMYB44 protein sequences in the same species presented high similarity with the protein sequences in *Actinidia eriantha* MYB44 and *Actinidia rufa* MYB73. Like those of the other species, the protein sequence in *Rhododendron vialii* MYB73 was highly similar (Supplementary Table S7) (Figure 6).

### 2.5. Expression of AaMYB44 in Response to Low-Temperature Treatment

**Figure 7:**
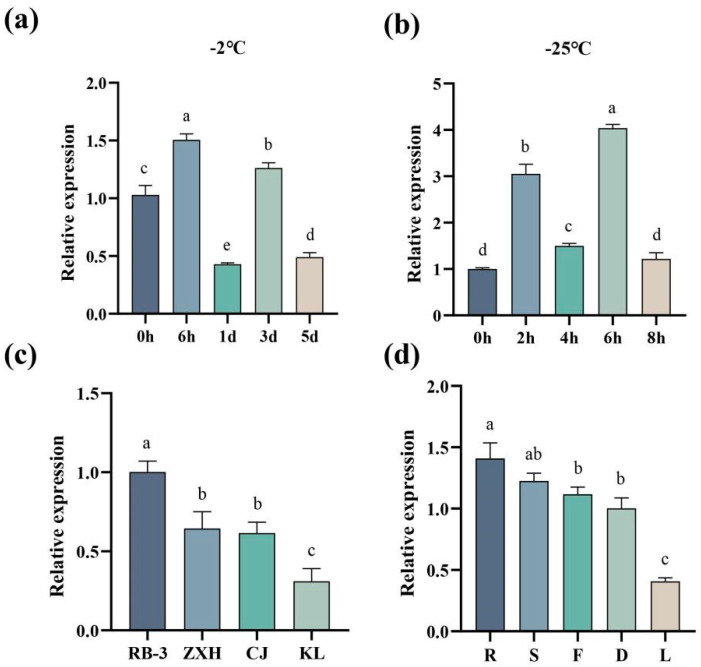
The RT‒PCR analysis of the *AaMYB44* expression in kiwifruit. (**a**) The expression of *AaMYB44* in shoots treated at −2 °C during the growth stage. (**b**) The expression of *AaMYB44* in dormant shoots treated at −25 °C. (**c**) The expression levels of *AaMYB44* in the different genotypes of *A. arguta*: Ruby-3: RB-3; ZhongXiaHong: ZXH; ChangJiang-1: CJ; and KuiLv: KL. (**d**) The tissue-specific analysis of the *AaMYB44* expression. R: root; S: growing shoot; F: flower; D: dormant shoot; and L: leaf. The different letters indicate significant differences, according to Duncan’s multiple range test at *p* < 0.05.

Potted KL plants in the growth stage were subjected to low-temperature treatment at −2 °C for 0 h, 6 h, 1 d, 3 d, or 5 d. Then, the expression of *AaMYB44* in the shoots was analyzed via quantitative real-time–polymerase chain reaction (qRT‒qPCR). The *AaMYB44* expression was highest in the KL shoots after 6 h of low-temperature treatment and first increased but then decreased from 1 d to 5 d (Figure 7a). Additionally, potted KL plants in the dormant stage were placed in a low-temperature treatment box at −25 °C for 0 h, 2 h, 4 h, 6 h, or 8 h. Then, the expression of *AaMYB44* in the shoots was analyzed via RT‒qPCR (Figure 7b). The expression of *AaMYB44* in the shoots first increased but then decreased at 8 h, peaking after 6 h of low-temperature treatment. The expression of *AaMYB44* tended to decrease in the dormant shoots of cold-sensitive genotypes (RB-3, ZXH, CJ, and KL) (Figure 7c), presenting the opposite trend observed in cold-tolerant shoots. The order of cold resistance in the four genotypes, from weak to strong, was as follows: RB-3, ZXH, CJ, and KL. The tissue-specific expression analysis results revealed that the highest expression level of *AaMYB44* was found in the roots during the growth stage, whereas the expression level of *AaMYB44* was lowest in the leaves. In particular, *AaMYB44* expression was lower in dormant shoots than in growing shoots (Figure 7d).

### 2.6. Subcellular Localization

**Figure 8:**
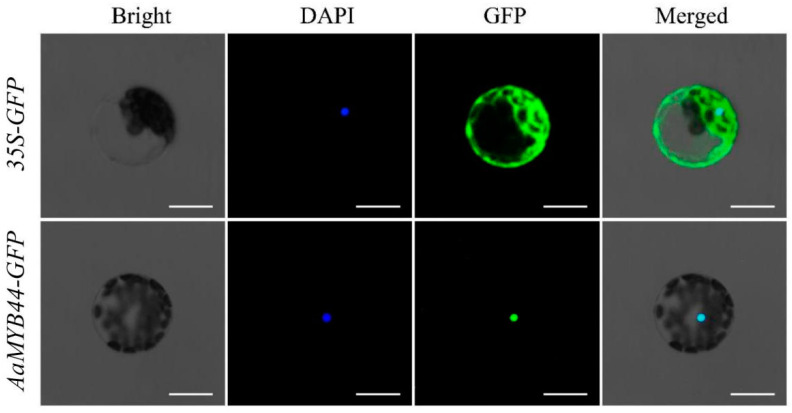
The subcellular localization of *AaMYB44*. The first (Bright), the second (DAPI), third (GFP), and fourth (Merged) panels represented bright fields, fluorescence, green fluorescence, and fusion of bright field, blue fluorescence, and green fluorescence, respectively. Scale bar = 10 μm.

The subcellular localization of AaMYB44 was examined upon its transient expression in the *A. thaliana.* We constructed an *AaMYB44*-green fluorescent protein (GFP) fusion vector and used an empty vector (EV) containing only GFP as a control. These plasmids, driven by the CaMV 35S promoter, were injected into *A. thaliana* protoplasts for transgene. The GFP fluorescence was ubiquitously distributed throughout the whole cell in those that received the control plasmid (Figure 8). In contrast, the GFP signal in the cells that received the recombinant plasmid was observed in the nuclei only. Overall, these results indicated that *AaMYB44* was located in the nucleus.

### 2.7. AaMYB44 Overexpression in A. thaliana Reduces Cold Tolerance

The *AaMYB44* open reading frame (ORF) driven by the constitutive CaMV 35S promoter was cloned and inserted into the pBI121 vector. The transgenic lines were obtained upon inflorescence infection, and gene expression was verified via RT‒qPCR. Three transgenic kiwifruit lines with high *AaMYB44* mRNA expression levels were selected for phenotypic and physiological analyses.

**Figure 9:**
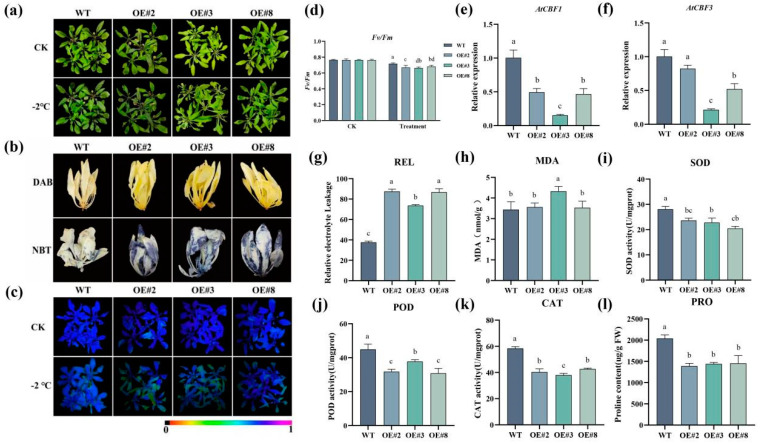
A characterization of tolerance mediated by *AaMYB44* in *Arabidopsis*. (**a**) The freezing phenotypes of the OE lines (#2, #3, and #8) and WT plants after cold stress (−2 °C for 4 h). (**b**) The 3,3’−DAB and NBT staining of the OE lines (#2, #3, and #8) and WT plants after cold stress (−2 °C for 4 h). (**c**,**d**) The *F_v_*/*F_m_* of *A. thaliana* plants after 4 h of low-temperature treatment. The color barcode is shown below the image. (**e**,**f**) The expression of *AtCBF1* and *AtCBF3* in *A. thaliana* under cold stress. (**g**) The REL values of the WT and OE plants. (**h**–**l**) The contents of MDA, SOD, POD, CAT, and PRO. CK indicates the *A. thaliana* plants before low-temperature treatment. The different letters indicate significant differences, according to Duncan’s multiple range test, *p* < 0.05.

No significant phenotypic differences were detected between the wild-type (WT) and transgenic plants under the same growth conditions. Three-week-old plants at similar growth stages were randomly selected and treated at −2 °C for 4 h. Compared with the WT plants, the transgenic plants presented more severe cold damage (Figure 9a). Histochemical staining of the plants with 3,3’-diaminobenzidine (3,3’-DAB) and nitroblue tetrazolium (NBT) at −2 °C revealed greater chlorophyll accumulation in the transgenic plants than in the WT plants (Figure 9b). Chlorophyll fluorescence imaging revealed that the chlorophyll content in the leaves of the WT plants was greater than that in the leaves of the transgenic plants after being subjected to low-temperature stress (Figure 9c). Due to low-temperature stress, the maximum quantum efficiency of photosystem II (PSII) (*F_v_*/*F_m_*) of both the WT and the transgenic plants decreased, but the *F_v_*/*F_m_* of the WT plants was greater than that of the transgenic plants (Figure 9d). Moreover, in response to low-temperature stress, the relative expression of *AtCBF1* and *AtCBF3* in the transgenic *A. thaliana* plants was lower than that in the WT plants (Supplementary Table S8) (Figure 9e,f). The relative electrolytic leakage (REL) values and the level of malondialdehyde (MDA), an important indicator of peroxidase activity in plants, were also measured. We found that the REL and MDA levels significantly increased in the transgenic plants under low-temperature stress (Figure 9g,h). Superoxide dismutase (SOD), peroxidase (POD), and catalase (CAT) play important roles in plant resistance to cold stress. We observed that the activities of SOD, POD, and CAT in the transgenic lines were significantly lower than those in the WT lines after 4 h of low-temperature treatment at −2 °C (Figure 9i,j,k). Proline (PRO) is an osmotic regulator needed by plants to manage abiotic stresses. After low-temperature treatment (−2 °C), the PRO content in the transgenic plants was significantly lower than that in the WT plants (Figure 9l). These results suggest that *AaMYB44*, as a negative regulator, reduces plant tolerance to cold stress by increasing the reactive oxygen species (ROS), REL, and MDA levels and reducing the activities of antioxidant enzymes and the contents of osmotic stress substances.

## 3. Discussion

Low temperatures can damage the ice coating inside plants and disturb their physiological processes, ultimately affecting their water status, photosynthetic properties, respiration, and response to cold stress, which can eventually cause death [27,28,29]. TFs play important roles in regulating plant responses to low-temperature stress [30]. MYB TFs are widely involved in plant responses to cold tolerance [31]. A total of 277 *MYB* genes were identified in kiwifruit, including nine that may respond to cold stress on the basis of transcriptome data [2]. Additionally, Sun reported that *A. arguta AaMYB5a* could help *AaMYC2a* bind to its target gene and increase the tolerance of *A. arguta* to cold stress [32]. In this study, 52 members of the *MYB* family were identified on the basis of the *A. arguta* transcriptomic data, all of which were expressed under low-temperature conditions. Conserved protein domains were identified in 52 members of the *AaMYB* gene family, wherein the *R2R3-MYB* family was found to respond strongly to low temperatures. In this study, via conservative structure domain and motif, structural analyses and evolutionary tree gene family identification were performed. The expression of genes in the low-temperature transcriptome data (Figure 5) and three *AaMYB* genes were induced by cold stress. In addition, their expression level increased significantly with continued cold stress duration. We identified M0t18803 (*AaMYB44*) in the *R2R3-MYB* subfamily as a candidate gene for further study. After the prediction of protein interactions, it was suggested that AaMYB44 and some TFs, such as MYC2, WRKR70, PYL8, et al., were interacting (Figure 4b). The ABA receptors PYL9 and PYL8 interaction plays an important role in regulating lateral root growth [33], although the ABA signal is involved in abiotic stress; however, there were no reports on abiotic stress. In our previous study, AaMYC2 could bind the promoter of *AaLAR* to accumulate PAs to increase cold tolerance in kiwifruit [32]. Therefore, we suspected that AaMYB44 might interact with AaMYCs in response to cold tolerance. The *AaMYB44* gene in *A. arguta* KL was down-regulated under low-temperature stress, whereas its expression level was greater in cold-sensitive genotypes; these findings suggest that *AaMYB44* may negatively regulate cold tolerance. MYB TFs play complex regulatory roles in response to abiotic conditions. *AtMyb73* causes hyperinduction of the *SOS1* and *SOS3* genes to increase the salt resistance of *A. thaliana* [34]. Additionally, overexpression of the *MbMYB4* gene in *A. thaliana* can increase the tolerance of transgenic plants to cold and drought stresses [35]. *XsMYB44* has a positive regulatory effect on the response of yellowhorn to combined stress by triggering stomatal closure to maintain water levels and modulating ROS homeostasis [36]. Moreover, *AhMYB44-11* plays a positive role in the drought tolerance of plants by increasing the transcript abundance of stress-related genes and the accumulation of osmolytes [15]. *AcMYB44* has been shown to play a positive regulatory role in the resistance of plants to drought [37]. Under drought conditions, the accumulation of *MYB44* and the decrease in *HDT4* levels could lead to increased expression of *H3K27ac* and drought response genes, thereby increasing the tolerance of the plant to drought stress [38].

However, in some plants, *MYB44* plays a negative regulatory role in the response to cold stress. For example, *VaMYB44* is a negative regulator of cold tolerance in grapes [39]. In our study, *AaMYB44* was overexpressed in *A. thaliana*, and the cold tolerance in the resulting transgenic lines was evaluated. The chlorophyll content represents the capacity of plants’ photosynthetic complex formation [40]. The chlorophyll structure is damaged in plants under low-temperature stress, which inhibits the biosynthesis of chlorophyll and leads to a significant reduction in the chlorophyll content [41]. The same result was observed in this study. After low-temperature treatment, fluorescence analysis revealed that the chlorophyll content in the transgenic *A. thaliana* plants was significantly lower than that in the WT plants, which implied that the chlorophyll content in the WT *A. thaliana* plants was not reduced as significantly as in the transgenic plants. *MYB* reportedly interacts with *CBF* genes in plants to allow their response to low temperatures. In Arabidopsis, MYB15 could negatively regulate the promoter of CBF genes and play a negative role in cold tolerance [42]. Therefore, in our study, we performed the relative expression of AtCBF1/2/3, and the results were consistent with previous reports under low-temperature treatment. The relative expression of Arabidopsis *AtCBF1* and *AtCBF3* in the transgenic plants was lower than that in the WT plants (Figure 9e,f). The MDA and REL levels can be used to assess the damage to the biofilms of plants under adverse conditions [43]. In this study, the MDA and REL levels of the overexpression lines were significantly greater than those of the WT. These results revealed that compared with those of the WT plants, the MDA and REL contents of the transgenic *A. thaliana* plants were abnormal. High ROS accumulation leads to oxidative stress and the inactivation of functional proteins, which harms cellular functions and biological activities [44]. In our study, the 3,3’-DAB and NBT staining results revealed that the content of ROS in the leaves of the WT plants was lower than that in the leaves of the AaMYB44-overexpressing plants. In addition, the ability of the WT plants to scavenge ROS at low temperatures was greater than that of the transgenic lines. SOD, POD, and CAT are commonly found in plant cells, and their activities are related to metabolic activity and cold resistance in the plant. These enzymes are important components of the enzymatic antioxidant system and can remove reactive oxygen free radicals, maintain membrane stability, and ensure the normal life activities of plants [45]. Moreover, compared with that of the WT plants, the MDA content, which is an indicator of the degree of membrane damage, decreased in AaMYB44-transformed plants, and MDA accumulation in WT plants was lower than that in plants expressing *AaMYB44* under oxidative stress. In this study, the levels of SOD, POD, and CAT in the transgenic plants were lower than those in the WT plants after low-temperature treatment, which suggested that the WT plants were able to scavenge various ROS. These results indicate that *AaMYB44* plays a negative regulatory role in the plant response to cold stress. These results provide a reference for the application of *AaMYB44* in fruit trees and the cultivation of cold-resistant varieties and lay a foundation for further research on the regulatory mechanism of *AaMYB44*. *AaMYB44* can also be used as an important candidate gene for cold resistance in kiwifruit and other plants. Although the results of this study cannot be directly applied to the actual production of kiwifruit in the short term, they could promote the accumulation of scientific knowledge and the progress of related technologies. Through continuous in-depth research, these theoretical foundations will eventually be translated into practical applications for crop production, plant breeding, and plant protection, in addition to other fields that are worth improving. Despite the limitations of this study, it is important to perform experiments similar to those outlined in this study, as they provide a solid foundation for future applications.

## 4. Materials and Methods

### 4.1. Plant Materials

The experimental materials used in this study were *A. arguta* RB-3, ZXH, CJ, and KL, which were planted in the kiwifruit resource nursery of the Zhengzhou Fruit Research Institute, Chinese Academy of Agricultural Sciences. Three-year-old potted plantlets with a height of approximately 140 cm, strong growth, no pests, and no diseases were selected. WT *A. thaliana* (Columbia-0) was also used and preserved by the Zhengzhou Fruit Research Institute, Chinese Academy of Agricultural Sciences.

### 4.2. Identification of the MYB Gene Families in A. arguta

We selected *MYB* genes from the full-length *A. arguta* transcriptome data, according to gene annotation, and then deleted incomplete and duplicate sequences by analyzing the CDSs of the genes. The structural domain of the MYB was verified based on the MYB-like DNA-binding domain. The isoelectric points and molecular weights of the MYB proteins were determined using ExPASy ProtParam (https://web.ExPASy.org/protparam/, accessed on 21 April 2024).

### 4.3. A Phylogenetic Tree Construction and Domain and Motif Distributions of the MYB Proteins in A. arguta

A phylogenetic tree was constructed using MEGA software (ver 7.0.26) and iTOL (https://itol.embl.de, accessed on 21 April 2024) on the basis of the AaMYB proteins. The AaMYB protein domain was identified using the Pfam database. The MEME Suite (https://meme-suite.org/meme/tools/meme, accessed on 21 April 2024) was used to predict the conserved motifs (with a maximum number of motifs of 10) in the AaMYB protein sequences. The results were visualized using TBtools software (ver 2.136).

### 4.4. RNA Extraction and Reverse Transcription

The experimental material was frozen in liquid nitrogen and then ground into powder in a mortar to extract total RNA. Total RNA was isolated using a Plant Total RNA purification kit (Vazyme, Beijing, China) following the manufacturer’s instructions. The RNA quality and concentration were determined using a NanoDrop spectrophotometer. The RNA samples were converted to cDNA using a single-stranded cDNA synthesis kit (BIOMAN, Beijing, China).

### 4.5. Isolation and Cloning of AaMYB44

The primers used were designed with Primer Premier 5.0. PCR was subsequently performed to clone the full-length sequence of *AaMYB44*. The following PCR amplification program was used: 95 °C for 3 min, 35 cycles of 95 °C for 15 s, 60 °C for 15 s, 72 °C for 45 min, and 72 °C for 5 min. The PCR products were examined via 1% agarose gel electrophoresis (200 V, 200 mA, 15 min), and target DNA fragments were recovered using the GeneJET Gel Extraction Kit (Thermo Fisher, Shanghai, China). The recovered products were attached to the T vector, according to the manufacturer’s instructions for the pMETM19-T Vector Cloning Kit, and the transformed *Escherichia coli* competent cells were placed in a constant-temperature incubator at 37 °C overnight to culture. White spots in the bacterial mixture were screened for PCR identification. The positive cloned bacterial mixture was used for DNA sequencing (Henan Shangya Biotechnology Co., Ltd., Zhengzhou, China). The results were sequentially spliced with DNAMAN8 and subsequently analyzed. First-generation Sanger sequencing was performed, and the target sequences were submitted to the NCBI for comparison.

### 4.6. AaMYB44 Expression Analysis

To analyze the expression of *AaMYB44* after low-temperature treatment, shoots in the growing stage and dormant stage were selected. The shoots in the growing stage were treated at −2 °C. Three grams of shoot tissue were collected from each plant at 0 h, 6 h, 1 d, 3 d, and 5 d. Dormant shoots were treated at −25 °C at 0 h, 2 h, 4 h, 6 h, and 8 h. To determine expression in different genotypes of *A. arguta*, RB-3, ZXH, CJ, and KL dormant shoots were collected in the winter. To determine tissue-specific expression, the shoots of growing and dormant plants, as well as the leaves, roots, and flowers of growing plants, were selected. Sampling was performed with three biological replicates of each material. Total RNA from all the materials was extracted using Vazyme’s Fastpure Universal Plant Total RNA Isolation Kit and BIOMAN RTIII All-in-One Mix with dsDNase reverse transcription after 5-fold dilution of the template cDNA. qPCR was performed using a Roche LightCycler^®^ 480 instrument (Roche, Basel, Switzerland)with the kiwifruit reference gene as the reference [46]. The expression pattern of *AaMYB44* in the shoots and leaves of kiwifruit was analyzed after low-temperature treatment. The total volume of the reaction mixture was 20 µL: 10 µL of 2× NovoStart^®^ SYBR SuperMix Plus (Novoprotein, Suzhou, China), 0.8 µL of 10 μmol L^−1^ Primer F, 0.8 µL of 10 μmol L^−1^ Primer R, 1 µL of cDNA, and 7.4 µL of ddH_2_O. The relative expression levels were calculated via the 2^−∆∆Ct^ method. All RT‒qPCR primers were designed using Premier 5.0 (Supplementary Table S8). Statistical analyses were performed in Excel (ver. 2016), GraphPad Prism (ver. 8.0.2), and SPSS software (ver. 22). T-tests and one-way ANOVA were used to analyze the data, and Duncan’s multiple comparisons were employed to compare differences between the samples. Values of *p* < 0.05 or *p* < 0.01 were considered to indicate statistical significance.

### 4.7. Bioinformatics Analysis

The AaMYB44 sequence alignment was performed using the NCBI BLAST tool (https://www.ncbi.nlm.nih.gov/Structure/cdd/wrpsb.cgi, accessed on 21 April 2024). The constructed domains of *AaMYB44* were predicted using the Pfam tool (https://www.ebi.ac.uk/interpro/entry/pfam, accessed on 21 April 2024). GeneDoc software (ver 2.7) was used to perform multiple protein sequence alignment and sequence alignment mapping. A phylogenetic tree was constructed using MEGA 7.0 software. The STRING (http://string-db.org/, accessed on 21 April 2024) was used to predict the interaction protein of AaMYB44, using *A. thaliana* as the reference genome. We set the following parameters: full String network was used as network type, evidence was chosen to draw the network edges, the active interaction was text mining, experiments, databases, co-expression, neighborhood, gene fusions, gene co-occurrence, the minimum required interaction score was medium confidence (0.400), and the max number of interactors to show was no more than 10 interactors.

### 4.8. Subcellular Localization of AaMYB44

In accordance with the sequence of *AaMYB44*, a series of PstI and BamHI restriction sites were added to the two ends of the *AaMYBB44* gene to amplify the gene fragment. Primer 5.0 software was used to design the primers (Supplementary Table S8). Hot polymerase (Vazyme, Beijing, China) was used to amplify the *AaMYB44* fragment, and the carrier enzyme digestion reagent PstI and the BamHI restriction enzyme (Thermo Fisher, Shanghai, China) were used to cut the genes and plasmids. A glue recovery kit (Magen, Guangzhou, China) was used to recover the enzyme-free products. The Seamless Cloning Kit (Universal, Hefei, China) reagents were used to generate the fragments and vectors. Afterward, the conjugation product was transformed into *E. coli* DH5α and 12 DH5α transformants were randomly selected from the conjugation system plate and inoculated on LBK media. After shaking at 37 °C and 250 rpm for 16 h (overnight), PCR amplification was performed via universal plasmid priming. One clone from the amplified positive band was used for plasmid extraction (using a Magen plasmid small-volume extraction kit) and sequencing. PCR amplification was performed with the PEGFP-N-3 reverse universal primer (Supplementary Table S8), and one clone from the amplified positive band was used for primer extraction (Magen plasmid small volume extraction kit) and sequencing. Once the sequences of the inserted fragments had been verified, we constructed an AaMYB44-GFP fusion vector and used an EV containing only GFP as a control. These plasmids, driven by the CaMV 35S promoter, were injected into *A. thaliana* protoplasts for transient transfection. Finally, the position of AaMYB44 was revealed using laser confocal microscopy.

### 4.9. A. thaliana Transformation

The *AaMYB44* ORF driven by the constitutive CaMV 35S promoter was cloned and inserted into pBI121. *Agrobacterium tumefaciens* (strain GV3101), harboring the 35S::AaMYB44 recombinant plasmid, was transformed into WT *A. thaliana* (Col-0) plants via the floral dip method. *A. thaliana* was subsequently transformed via inflorescence infection. The eukaryotic binary expression vector AaMYB44::GFP was constructed, and plasmid DNA was extracted and transformed into *Agrobacterium* GV3101 via electroporation. After transformed *A. tumefaciens* was obtained, the bacteria were cultured until the OD of the bacterial mixture reached 0.5–1.5. The bacteria were collected via centrifugation and then resuspended in an osmotic buffer containing sucrose (5%) and Silwet L-77 (0.01%). The grown horn fruits from *A. thaliana* seedlings in the reproductive growth stage were removed, and the samples were immersed in a dye solution for approximately 1.5 min. The stained *A. thaliana* seedlings were covered with black film for 24 h in the dark to maintain sufficient water levels. Owing to their long flowering period, the *A. thaliana* seedlings were immersed 2–3 times. T0 seeds were collected after the natural cracking of the horn fruit. The positive plants were screened with a screening medium, and seeds of the T1 generation were collected after the positive plants were seeded again. Homozygote T3 seeds were obtained after three successive sowings. Three T3-positive transgenic lines were selected for further verification of gene function. Primers for the AaMYB44-1842-transformed plants were then designed using Primer 5.0 (Supplement Table S8). The transformed plants were identified via PCR and RT‒qPCR to verify the successful introduction of the transgene. Three positive strains of transgenic kiwifruit with high levels of *AaMYB44* mRNA expression (OE#2, OE#3, and OE#8) were selected for molecular and phenotypic analyses.

### 4.10. Cold Treatment of Transgenic A. thaliana and Evaluation of Plant Cold Tolerance

WT *A. thaliana* (control) and T3 generation transgenic plants (OE#2, OE#3, and OE#8) were seeded in a basin that was 9 cm in diameter and 15 cm deep and placed in an incubator (25 °C temperature; 12 h light, 12 h dark; 60% humidity). When all the *A. thaliana* plants had four leaves, they were transferred to new pots, with five vigorous and evenly sized *A. thaliana* plants in each pot. The WT and three transgenic *A. thaliana* lines were transplanted into 20 pots each and placed in an incubator.

To evaluate physiology, 3-week-old *A. thaliana* plants from the WT and transgenic lines were subjected to treatment at −2 °C for 2 h. Afterward, the activities of SOD, POD, and CAT and the contents of PRO and MDA were determined, as described in the ELISA kit manuals. REL was measured according to the method described by Sun [47]. The accumulation of hydrogen peroxide (H_2_O_2_) and superoxide (O^2−^) under cold stress was determined via 3,3’-DAB and NBT staining methods [48,49]. To measure chlorophyll fluorescence, whole plants were imaged with an IMAGING-PAM chlorophyll fluorometer (Walz, Effeltrich, Germany); the plants were placed in a dark room for 30 min prior to imaging. The *F_v_*/*F_m_* was measured using Imaging Win-GegE software (ver 2.56zn) [50].

## 5. Conclusions

In conclusion, this study used the transcriptomic data of kiwifruit under low-temperature stress to identify 52 *MYB* genes, and we analyzed the *MYB* families of *A. arguta* that respond to low temperatures. We found that *AaMYB44* is a TF located in the nucleus, and the expression of *AaMYB44* is lower in cold-tolerant *A. arguta.* The overexpression of *AaMYB44* in *A. thaliana* results in decreased cold resistance, indicating that it has a negative role in the regulation of cold tolerance. In addition, this study provides a theoretical basis for the molecular cultivation of kiwifruit and other plants for cold resistance.

## Data Availability

No new data were created or analyzed in this study. Data sharing is not applicable to this article.

## Supplementary Materials

The following supporting information can be downloaded at: https://www.mdpi.com/article/10.3390/plants13223126/s1, Table S1: The genes used in the phylogenetic tree compared with *A. chinensis*. Table S2: *MYB* gene information of *Arabidopsis thaliana* used in phylogenetic tree. Table S3: Classification information of 52 *MYB* genes in the phylogenetic tree of *A. arguta*. Table S4: The chemical characteristics of the AaMYB family in *A. arguta*. Table S5: The FPKM value of *AaMYB* genes from the two *A. arguta* under cold stress based on transcriptomic data. Table S6: Genebank accessions used in Alignment sequences in Figure 5. Table S7: Genebank accessions used in phylogenetic tree. Table S8: Primer sequences used in this study.

## References

[1] Lin M.M., Sun S.A., Qi X.J., Wang R., Fang J.B. (2020). Advances in research on cold resistance in kiwifruit. J. Fruit Sci..

[2] Mao K., An M., Wang H., Wang S., Lü W., Guo Y., Li J., Li G. (2023). Identification and Low Temperature Expression Analysis of MYB Transcription Factor Family in Kiwifruit. Acta Hortic. Sin..

[3] Hartmann T., Scheiner J., Stein L., King A., Feagely S. (2021). Young Field-grown Kiwifruit Plants’ Response to Early Autumn Frost Injury in Texas. HortTechnology.

[4] Testolin R., Costa G., Comuzzo G., Galliano A., Vittone F., Mescalchin M., Gobber M., Michelotti F., Trentini G., Crivello V. (1994). Kiwifruit harvesting and the danger of frost La raccolta dell’actinidia e i pericoli di gelate. Inf. Agrar..

[5] Ebrahimi Y., Jorshari H., Lashtneshaii K., Homam K. Frost Damage on Kiwifruit in Iran. Proceedings of the 7th International Symposium on Kiwifruit.

[6] Gao P., Gao B., Feng Z., Wu J. (2023). Cloning and Cd-resistant Analysis of PsWRKY40 in Potentilla sericea. Acta Hortic. Sin..

[7] Baillo E.H., Kimotho R.N., Zhang Z., Xu P. (2019). Transcription Factors Associated with Abiotic and Biotic Stress Tolerance and Their Potential for Crops Improvement. Genes.

[8] Shi X.-Y., Li T., Wang H.-Y., Zhnag R.-Q., Cao S.-J., Zhang L.-L., Yao J.-G., Liu J.-F. (2023). Research progress of tomato MYB transcription factor. Chin. Melon Veg..

[9] Wang X., Niu Y., Zheng Y. (2021). Multiple functions of MYB transcription factors in abiotic stress responses. Int. J. Mol. Sci..

[10] Katiyar A., Smita S., Lenka S.K., Rajwanshi R., Chinnusamy V., Bansal K. (2012). Genome-wide classification and expression analysis of MYB transcription factor families in rice and Arabidopsis. BMC Genom..

[11] Wang F., Yang F., Zhu D., Saniboere B., Zhou B., Peng D. (2023). MYB44 plays key roles in regulating plant responses to abiotic and biotic stress, metabolism, and development. J. Plant Biochem. Biotechnol..

[12] Yang A., Dai X., Zhang W.H. (2012). A R2R3-type MYB gene, OsMYB2, is involved in salt, cold, and dehydration tolerance in rice. J. Exp. Bot..

[13] Li M., Cai Y., Yang X., Huang S., Li J., Tan Q., Qiu W., Fang W. (2021). Cloning and Expression Analysis of MYB Gene AcoMYB1 in Pineapple (*Ananas comosus*). Chin. J. Trop. Crops.

[14] An J.-P., Wang X.-F., Zhang X.-W., Xu H.-F., Bi S.-Q., You C.-X., Hao Y.-J. (2020). An apple MYB transcription factor regulates cold tolerance and anthocyanin accumulation and undergoes MIEL1-mediated degradation. Plant Biotechnol. J..

[15] Liu Y., Shen Y., Liang M., Zhang X., Xu J., Shen Y., Chen Z. (2022). Identification of Peanut AhMYB44 Transcription Factors and Their Multiple Roles in Drought Stress Responses. Plants.

[16] Agarwal M., Hao Y., Kapoor A., Dong C.H., Fujii H., Zheng X., Zhu J.K. (2006). A R2R3 type MYB transcription factor is involved in the cold regulation of CBF genes and in acquired freezing tolerance. J. Biol. Chem..

[17] Gao F., Yao H., Zhao H., Zhou J., Luo X., Huang Y., Li C., Chen H., Wu Q. (2016). Tartary buckwheat FtMYB10 encodes an R2R3-MYB transcription factor that acts as a novel negative regulator of salt and drought response in transgenic Arabidopsis. Plant Physiol. Biochem..

[18] Ampomah-Dwamena C., Thrimawithana Amali H., Dejnoprat S., Lewis D., Espley Richard V., Allan Andrew C. (2019). A kiwifruit (*Actinidia deliciosa*) R2R3-MYB transcription factor modulates chlorophyll and carotenoid accumulation. New Phytol..

[19] Wang R., Bourke P.M., Li S., Lin M., Sun L., Gu H., Li Y., Visser R.G.F., Qi X., Maliepaard C. (2024). QTL mapping of fruit quality traits in tetraploid kiwiberry (*Actinidia arguta*). Hortic. Plant J..

[20] Yu M., Man Y., Wang Y. (2019). Light- and Temperature-Induced Expression of an R2R3-MYB Gene Regulates Anthocyanin Biosynthesis in Red-Fleshed Kiwifruit. Int. J. Mol. Sci..

[21] Horák M., Šnurkovič P., Ondrášek I., Balík J., Srilaong V. (2019). Comparison of some physico-chemical parameters of kiwiberry (*Actinidia arguta*) cultivars from a cold climate. Folia Hortic..

[22] Huang G. (2020). Development status and problems of Actinidia arguta industry. North. Fruits.

[23] Sun S., Lin M., Qi X., Chen J., Gu H., Zhong Y., Sun L., Muhammad A., Bai D., Hu C. (2021). Full-length transcriptome profiling reveals insight into the cold response of two kiwifruit genotypes (*A. arguta*) with contrasting freezing tolerances. BMC Plant Biol..

[24] Lu X.M., Yu X.F., Li G.Q., Qu M.H., Wang H., Liu C., Man Y.-P., Jiang X.-H., Li M.-Z., Wang J. (2024). Genome assembly of autotetraploid Actinidia arguta highlights adaptive evolution and dissects important economic traits. Plant Commun..

[25] Liu Y., Wang R., Yu J., Huang S., Zhang Y., Wei H., Wei Z. (2023). Genome-Wide Identification and Characterization of Auxin Response Factor (ARF) Gene Family Involved in Wood Formation and Response to Exogenous Hormone Treatment in Populus trichocarpa. Int. J. Mol. Sci..

[26] Liu C., Xie T., Chen C., Luan A., Long J., Li C., Ding Y., He Y. (2017). Genome-wide organization and expression profiling of the R2R3-MYB transcription factor family in pineapple (*Ananas comosus*). BMC Genom..

[27] Yu F., Zhi Q. (2009). The harm of low temperature to plants and the cold resistance of plants. Anim. Husb. Feed Sci..

[28] Hassan M.A., Xiang C., Farooq M., Noor M. (2021). Cold Stress in Wheat: Plant Acclimation Responses and Management Strategies. Front. Plant Sci..

[29] Saleem M., Fariduddin Q., Janda T. (2021). Multifaceted role of salicylic acid in combating cold stress in plants: A review. J. Plant Growth Regul..

[30] Mohammsd M., Beenish F., Mohammad A., Yan C., Beirui W., Yan Q. (2022). Plant low-temperature stress: Signaling and response. Agronomy.

[31] Dong J., Cao L., Zhang X., Zhang W., Yang T., Zhang J., Che D. (2021). An R2R3-MYB Transcription Factor RmMYB108 Responds to Chilling Stress of Rosa multiflora and Conferred Cold Tolerance of Arabidopsis. Front. Plant Sci..

[32] Sun S., Qi X., Zhang Z., Sun L., Wang R., Li Y., Chen J., Gu H., Fang J., Lin M. (2024). A structural variation in the promoter of the leucoanthocyanidin reductase gene AaLAR1 enhances freezing tolerance by modulating proanthocyanidin accumulation in kiwifruit (*Actinidia arguta*). Plant Cell Environ..

[33] Xing L., Zhao Y., Gao J., Xiang C., Zhu J.K. (2016). The ABA receptor PYL9 together with PYL8 plays an important role in regulating lateral root growth. Sci. Rep..

[34] Kim J.H., Nguyen N.H., Jeong C.Y., Nguyen N.T., Hong S.-W., Lee H. (2013). Loss of the R2R3 MYB, AtMyb73, causes hyper-induction of the SOS1 and SOS3 genes in response to high salinity in Arabidopsis. J. Plant Physiol..

[35] Yao C., Li X., Li Y., Yang G., Liu W., Shao B., Zhong J., Huang P., Han D. (2022). Overexpression of a Malus baccata MYB Transcription Factor Gene MbMYB4 Increases Cold and Drought Tolerance in Arabidopsis thaliana. Int. J. Mol. Sci..

[36] Li J., Zhao S., Yu X., Du W., Li H., Sun Y., Ruan C. (2021). Role of Xanthoceras sorbifolium MYB44 in tolerance to combined drought and heat stress via modulation of stomatal closure and ROS homeostasis. Plant Physiol. Biochem..

[37] Qi Y., Wang L.H., Zhang Y.B., Cheng H., Liu B., Liu Y.S. (2022). Functional characterization of the AcMYB44 transcription factor of kiwifruit (*Actinidia chinensis*) in response to drought stress. Chin. J. Appl. Environ. Biol..

[38] Zhao B., Shao Z., Wang L., Zhang F., Chakravarty D., Zong W., Dong J., Song L., Qiao H. (2022). MYB44-ENAP1/2 restricts HDT4 to regulate drought tolerance in Arabidopsis. PLoS Genet..

[39] Zhang H., Hu Y., Gu B., Cui X., Zhang J. (2022). VaMYB44 transcription factor from Chinese wild Vitis amurensis negatively regulates cold tolerance in transgenic *Arabidopsis thaliana* and *V. vinifera*. Plant Cell Rep..

[40] Wei Z.Z., Hu K.D., Zhao D.L., Tang J., Huang Z.Q., Jin P., Li Y.-H., Han Z., Hu L.-Y., Yao G.-F. (2020). MYB44 competitively inhibits the formation of the MYB340-bHLH2-NAC56 complex to regulate anthocyanin biosynthesis in purple-fleshed sweet potato. BMC Plant Biol..

[41] Ma X., Yu Y.N., Jia J.H., Li Q.H., Gong Z.H. (2022). The pepper MYB transcription factor CaMYB306 accelerates fruit coloration and negatively regulates cold resistance. Sci. Hortic..

[42] Shi Y., Ding Y., Yang S. (2018). Molecular Regulation of CBF Signaling in Cold Acclimation. Trends Plant Sci..

[43] Sun S., Hu C., Qi X., Chen J., Zhong Y., Muhammad A., Lin M., Fang J. (2021). The AaCBF4-AaBAM3.1 module enhances freezing tolerance of kiwifruit (*Actinidia arguta*). Hortic. Res..

[44] Huang X.S., Wang W., Zhang Q., Liu J.H. (2013). A basic helix-loop-helix transcription factor, PtrbHLH, of Poncirus trifoliata confers cold tolerance and modulates peroxidase-mediated scavenging of hydrogen peroxide. Plant Physiol..

[45] Liu C., Yu Y., Chen Y., Yang Z. (2015). Water stress on tomato leaves at seedling stage Effects of protective enzyme activity and plant morphology. North. Hortic..

[46] Yu D., Zhang G., Chen J., Xu X., Li L., Dong P., Gou L., Yang H. (2023). Construction of Arabidopsis plants with AtIDD4 gene overexpression and its effect on growth and development. Southwest China J. Agrocultural Sci..

[47] Sun S., Lin M., Qi X., Zhao J., Meng X., Fang J. (2019). Determination of Semi-lethal Temperature of Kiwifruit by Electrolyte Leakage Method. North. Hortic..

[48] Thordal-Christensen H., Zhang Z., Wei Y., Collinge D.B. (1997). Subcellular localization of H_2_O_2_ in plants. H_2_O_2_ accumulation in papillae and hypersensitive response during the barley—Powdery mildew interaction. Plant J..

[49] Jabs T., Dietrich R.A., Dangl J.L. (1996). Initiation of runaway cell death in an Arabidopsis mutant by extracellular superoxide. Science.

[50] Su L., Dai Z., Li S., Xin H. (2015). A novel system for evaluating drought cold tolerance of grapevines using chlorophyll fluorescence. BMC Plant Biol..

