# Supplementary material for: Overexpression of the Kiwifruit Transcription Factor AaMYB44 Decreases the Cold Tolerance in Arabidopsis thaliana
Source: Plants (Basel). 2024 Nov 6;13(22):3126. doi: 10.3390/plants13223126 (PMC11597321; doi:10.3390/plants13223126)
Supplement: Supplementary file 1 [file plants-13-03126-s001.zip › plants-3216874-supplementary.pdf]

Supplementary Table S1. The genes used in the phylogenetic tree compared with *A. chinensis*

MYB ID abbreviation	MYB ID of *A. arguta* transcriptome	MYB ID of ('Hong yang' V2 version)
M0t_2997	MWXS20249_01mi_transcript_2997	Ach00g246011
M0t_14988	MWXS20249_01mi_transcript_14988	Ach05g363981
M0t_17266	MWXS20249_01mi_transcript_17266	Ach25g141911
M0t_17547	MWXS20249_01mi_transcript_17547	Ach05g363981
M0t_9751	MWXS20249_01mi_transcript_9751	Ach00g139181
M0t_4174	MWXS20249_01mi_transcript_4174	Ach13g190451
M0t_12366	MWXS20249_01mi_transcript_12366	Ach00g218481
M0t_18560	MWXS20249_01mi_transcript_18560	Ach15g243861
M0t_3930	MWXS20249_01mi_transcript_3930	Ach00g246011
M0t_15319	MWXS20249_01mi_transcript_15319	Ach15g243861
M0t_15713	MWXS20249_01mi_transcript_15713	Ach15g243861
M0t_17347	MWXS20249_01mi_transcript_17347	Ach03g188021
M0t_15156	MWXS20249_01mi_transcript_15156	Ach25g141911
M0t_4442	MWXS20249_01mi_transcript_4442	Ach13g190451
M0t_5416	MWXS20249_01mi_transcript_5416	Ach13g190451
M0t_4375	MWXS20249_01mi_transcript_4375	Ach00g096741
M0t_4412	MWXS20249_01mi_transcript_4412	Ach00g478211
M0t_15196	MWXS20249_01mi_transcript_15196	Ach03g188021
M0t_17493	MWXS20249_01mi_transcript_17493	Ach07g006361
M0t_12746	MWXS20249_01mi_transcript_12746	Ach00g139181
M0t_7919	MWXS20249_01mi_transcript_7919	Ach17g471351
M0t_4247	MWXS20249_01mi_transcript_4247	Ach00g096741
M0t_21980	MWXS20249_01mi_transcript_21980	Ach21g372141
M0t_15647	MWXS20249_01mi_transcript_15647	Ach11g049881
M0t_16530	MWXS20249_01mi_transcript_16530	Ach00g384141
M0t_3624	MWXS20249_01mi_transcript_3624	Ach00g246011
M0t_7499	MWXS20249_01mi_transcript_7499	Ach17g471351
M0t_3515	MWXS20249_01mi_transcript_3515	Ach21g371541
M0t_14875	MWXS20249_01mi_transcript_14875	Ach00g035581
M0t_18922	MWXS20249_01mi_transcript_18922	Ach03g188021
M0t_1895	MWXS20249_01mi_transcript_1895	Ach21g371551

MYB ID abbreviation	MYB ID of *A. arguta* transcriptome	MYB ID of ('Hong yang' V2 version)
M0t_3859	MWXS20249_01mi_transcript_3859	Ach00g478211
M0t_11186	MWXS20249_01mi_transcript_11186	Ach00g218481
M0t_22110	MWXS20249_01mi_transcript_22110	Ach00g328791
M0t_14648	MWXS20249_01mi_transcript_14648	Ach11g049881
M0t_18123	MWXS20249_01mi_transcript_18123	Ach03g188021
M0t_17065	MWXS20249_01mi_transcript_17065	Ach26g108411
M0t_1138	MWXS20249_01mi_transcript_1138	Ach21g371551
M0t_14411	MWXS20249_01mi_transcript_14411	Ach06g233451
M0t_21321	MWXS20249_01mi_transcript_21321	Ach21g372141
M0t_13779	MWXS20249_01mi_transcript_13779	Ach00g139181
M0t_17598	MWXS20249_01mi_transcript_17598	Ach05g363981
M0t_18803	MWXS20249_01mi_transcript_18803	Ach00g471961
M0t_19302	MWXS20249_01mi_transcript_19302	Ach07g006361
M0t_17312	MWXS20249_01mi_transcript_17312	Ach15g001361
M0t_13866	MWXS20249_01mi_transcript_13866	Ach15g243861
M0t_17275	MWXS20249_01mi_transcript_17275	Ach06g183551
M0t_14684	MWXS20249_01mi_transcript_14684	Ach05g363981
M0t_19532	MWXS20249_01mi_transcript_19532	Ach00g328791
M0t_4984	MWXS20249_01mi_transcript_4984	Ach19g198661
M0t_1554	MWXS20249_01mi_transcript_1554	Ach00g044311
M0t_1801	MWXS20249_01mi_transcript_1801	Ach21g371551

Supplementary Table S2. ***MYB*** **gene information of** ***Arabidopsis thaliana*** **used in phylogenetic tree**

Abbreviation gene name	Gene name	NCBI ID
AtM109	AtMYB109	NP_187534.1
AtM073	AtMYB073	NP_187534.1
AtM064	AtMYB064	NP_195443.1
AtM101	AtMYB101	NP_180805.1
AtM058	AtMYB058	NP_173098.1
AtM066	AtMYB066	NP_196979.1
AtM036	AtMYB036	NP_200570.1

Supplementary Table S3. Classification information of 52 ***MYB*** **genes in the phylogenetic tree of** ***A. arguta*.**

Group A	Group B	Group C	Group D	Group E	Group F	Group G
M0t_14411	M0t_1801	M0t_4412	M0t_14875	M0t_4984	M0t_16530	M0t_18123
M0t_9751	M0t_1138	M0t_3859	M0t_18803	M0t_1554	M0t_7919	M0t_18922
M0t_13779	M0t_1895	M0t_17065	M0t_19302	M0t_4174	M0t_7499	M0t_17347
M0t_12746	M0t_3515	M0t_21980	M0t_17493	M0t_5416	M0t_4375	M0t_15196
	M0t_2997	M0t_21321	M0t_22110	M0t_4442	M0t_4247	M0t_17275
	M0t_3930		M0t_19532		M0t_15731	M0t_11186
	M0t_3624		M0t_14988		M0t_18560	M0t_12366
			M0t_17547		M0t_13866	M0t_14648
			M0t_14684		M0t_15319	M0t_15647
			M0t_17598			M0t_17312
						M0t_17266
						M0t_15156

Supplementary Table S4. The chemical characteristics of the AaMYB family in *A. arguta*.

Gene ID	Amino acids	Molecular weight	Theoretical pI	Instability index
M0t_12746	399	44756.5	8.3	63.31
M0t_13779	399	44756.5	8.3	63.31
M0t_14411	334	37884.22	8.7	54.54
M0t_1801	766	84120.46	5.91	52.22
M0t_1895	766	84242.6	5.82	54.67
M0t_9751	399	44800.5	8.02	63.21
M0t_1138	766	84120.46	5.91	52.22
M0t_1554	813	89873.65	5.89	49.45
M0t_2997	650	74211.64	8.27	54.1
M0t_3515	650	74233.66	7.85	53.41
M0t_3624	650	74181.62	8.27	54.46
M0t_3930	650	74211.64	8.27	54.1
M0t_4984	623	68340.43	5.4	47.14
M0t_17065	296	34050.76	8.59	67.66
M0t_21321	274	30270.34	8.78	58.43
M0t_21980	274	30256.31	8.78	60.41
M0t_14875	251	29601.12	6.87	78.95
M0t_3859	640	67454.37	6.36	43.08
M0t_4412	636	67098.91	6.26	42.32
M0t_4174	714	78616.59	8.6	64.63
M0t_4442	712	78424.5	8.7	65.01
M0t_5416	712	78411.42	8.46	64.55
M0t_14988	333	36186.41	8.94	67.17
M0t_17493	270	29770.24	6.67	66.98
M0t_17547	333	36145.4	8.94	65.68
M0t_17598	333	36240.5	8.94	67.17
M0t_19302	270	29770.24	6.67	66.98
M0t_19532	270	29270.16	9.19	55
M0t_22110	270	29270.16	9.19	55
M0t_14684	333	36240.5	8.94	67.17
M0t_18803	278	30742.54	9.22	56.71
M0t_15156	340	37108.28	6.76	58.42

Gene ID	Amino acids	Molecular weight	Theoretical pI	Instability index
M0t_15647	350	38127.9	7.18	54.4
M0t_17266	295	32635.44	6.35	56.47
M0t_17275	275	30235.88	8.71	60.66
M0t_17312	342	36951.95	6.78	58.43
M0t_14648	262	28571.14	8.99	57.26
M0t_15196	308	33809.14	8.86	39.47
M0t_17347	279	30511.58	9.37	35.98
M0t_18123	306	33590.99	8.83	38.72
M0t_18922	306	33590.99	8.83	38.72
M0t_12366	295	33930.98	7.91	63.42
M0t_11186	295	33948.97	7.27	64.91
M0t_16530	300	32468.51	6.32	54.72
M0t_15713	317	34563.6	5.8	46.88
M0t_18560	317	34568.63	5.8	47.86
M0t_4247	673	74192.38	6.12	43.68
M0t_4375	673	74249.53	6.3	43.58
M0t_7499	403	44779.3	7.32	57.4
M0t_7919	408	45181.89	8.41	55.98
M0t_15319	317	34536.57	5.8	48.1
M0t_13866	317	34536.57	5.8	48.1

Supplementary Table S5. The FPKM value of *AaMYB* genes from the two *A. arguta* under cold stress based on transcriptomic data

ID	FPKM																							
	K0-1	K0-2	K0-3	K1-1	K1-2	K1-3	K4-1	K4-2	K4-3	K7-1	K7-2	K7-3	R0-1	R0-2	R0-3	R1-1	R1-2	R1-3	R4-1	R4-2	R4-3	R7-1	R7-2	R7-3
M0t_2997	0	0	0	0.04	0	0	0	0	0	0	0	0	6.25	7.81	5.21	5.52	3.84	7.08	6.26	4.98	6.6	4.75	4.22	2.92
M0t_14988	0.82	1.35	1.32	0.14	0	0.34	0.87	0.43	1.12	0.79	0.88	0.73	12.7	321.15	297.16	238.66	235.32	242.46	261.11	272.7	250.71	258.76	293.35	256.01
M0t_17266	0.19	0.18	0.73	0.19	0.24	0.73	0.73	0.82	0.64	0.15	0.88	0.18	7.39	9.5	9.33	11.46	11.01	14.29	16.72	15.31	16.33	15.71	13.73	15.82
M0t_17547	0.42	0.57	1.05	0	0.59	0.55	0.61	0	0.89	0.92	2.14	1.38	152.4	151.21	155.82	125.82	117.83	125.15	141.71	146.98	143.99	131.97	144.5	126.24
M0t_9751	1.68	1.11	1.44	1.66	1.22	0.98	2.59	1.39	1.25	2.21	2.53	2.3	18.83	21.63	16.51	26.1	25.68	24.03	36.19	36.92	36.37	29.42	32.86	27.26
M0t_4174	0.72	1.1	1.01	0.68	1.45	1.18	1.04	1.46	1.3	0.98	1.02	1.24	7.71	8.29	7.08	7.62	6.43	7.99	6.72	7.81	5.78	8.2	8.27	7.43
M0t_12366	4.35	3.39	3.13	3.36	2.25	2.48	2.99	2.57	2.18	2.15	3.48	4.42	33.53	34.76	32.85	36.31	37.88	38.36	40.43	37.95	41.57	34.1	38.37	37.82
M0t_18560	1.73	2.11	1.52	3.05	2.49	3.75	1.52	1.73	1.05	1.23	2.79	2.03	31.68	32.69	30.54	44.29	42.61	42.99	37.04	38.65	39.41	44.24	44.86	37.8
M0t_3930	3.54	3.2	4.18	2.06	2.59	2.3	1.96	2.27	2.43	2.14	2.38	2.24	10.37	12.02	13.35	12.08	11.39	10.25	11.59	12.81	12.49	12.69	14.81	15.61
M0t_15319	2.04	2.27	1.59	3.37	2.63	2.18	3.77	3.8	4.13	2.36	3.19	3.8	24.21	23.02	24.45	29.95	30.37	32.33	31.15	32.89	29.56	28.9	35.52	32.15
M0t_15713	2.65	4.14	4	3.93	2.68	2.98	2.88	3.68	3.52	1.46	2.47	2.86	26.66	30.44	27.19	33.62	32.39	37.59	30.6	34.08	34.6	41.21	36.1	35.61
M0t_17347	3.64	4.81	3.28	2.97	3.5	4.86	3.15	2.94	3.98	3.98	3.23	3.03	0.54	1.24	0.61	0.69	0.61	0.66	0.84	0.59	1.1	0.75	1.65	1.08
M0t_15156	2.38	3.05	4.55	2.77	3.67	4.03	3.08	4.02	4.37	2.91	3.49	2.62	5.74	7.14	6.79	10.37	10.68	10.74	12.3	13.95	11.92	13.47	14.48	14.98
M0t_4442	4.94	4.1	4.1	3.88	3.82	3.11	4.38	3.56	3.79	4.07	3.68	3.48	1.31	1.55	0.94	1.22	1.45	0.79	1.04	1.71	1.1	1.37	1.34	1.74
M0t_5416	5.63	5.43	4.97	5.32	4.66	5.45	4.94	6.9	5.63	4.51	6.04	5.77	1.41	1.82	1.78	1.13	1.22	2.48	2.1	1.28	1.63	1.57	1.11	1.74
M0t_4375	6.23	5.31	5.43	5.24	5.9	5.16	3.94	4.9	3.78	3.99	4.91	4.47	11.03	11.63	11.52	11.63	10.22	13.34	9.79	8.66	13.07	11.52	14.17	12.11

ID	FPKM																							
	K0-1	K0-2	K0-3	K1-1	K1-2	K1-3	K4-1	K4-2	K4-3	K7-1	K7-2	K7-3	R0-1	R0-2	R0-3	R1-1	R1-2	R1-3	R4-1	R4-2	R4-3	R7-1	R7-2	R7-3
M0t_4412	5.74	6.01	5.25	6.37	7.31	6.45	5.69	6.31	6.26	3.54	5.54	4.47	9.44	9.26	11.94	10.96	6.9	10.22	10.63	11.25	9.22	11.44	11.64	10.58
M0t_15196	7.72	4.14	3.8	7.36	7.44	7.24	6.99	6.9	7.3	7.28	7.1	6.94	13.57	12.37	8.49	14.6	11.2	6.49	7.01	12.97	5.02	11.43	7.32	13.49
M0t_17493	4.08	3.13	3.13	7.77	8.79	1.02	4.26	5.26	5.7	5.24	4.75	5.13	3.06	2.59	13.07	2.82	7.64	4.64	8.1	0	3.99	7.22	5.89	8.28
M0t_12746	15.79	16.46	13.64	12.12	10.25	15.2	5.49	7.92	24.38	9.5	16.97	35.94	27.39	31.35	28.47	33.68	32.97	31.71	29.81	28.35	27.33	28.16	32.76	33.84
M0t_7919	11.33	9.92	11.51	10.26	10.53	9.93	9.33	8.38	8.39	9.17	8.92	11.28	2.76	2.46	2.34	3.26	3.8	3.11	3.42	3.74	3.08	2.59	3.64	2.51
M0t_4247	10.94	10.09	12.17	11.99	11.03	11.06	10.56	10.15	10.84	8.52	10.31	8.48	30.4	32.22	23.1	34.36	40.2	32.97	36.27	40.66	30.94	37.32	35.84	33.6
M0t_21980	13.72	8.97	11.34	10.13	11.48	14.84	13.5	14.08	11.64	14.4	17.34	16	41.91	39.65	39.64	48.79	44.83	48.05	41.97	46.53	45.28	45.98	48.45	42.66
M0t_15647	12.4	13.41	11.94	12.52	11.71	12.14	13.62	12.7	11.31	12.3	17.12	14.83	0	0	0	0	0	0	0	1.11	2.43	0	1.29	0
M0t_16530	18.42	11.5	16.28	18.13	13.89	13.95	9.84	11.23	18.73	15.64	15.6	12.15	4.29	4.69	3.14	3.34	3.33	3.11	3.23	5.43	3.94	3.38	4.92	4.71
M0t_3624	14.75	14.8	12.51	15.16	14.03	13.58	14.97	14.09	14.3	14.55	15.81	15.01	0.56	0.45	0.45	0.79	0.44	0.52	0.34	0.55	0.21	0.23	0.54	0.76
M0t_7499	15.4	15.35	12.26	13.06	15.93	5.42	13.22	13.29	12.93	16.7	15.65	15.19	0.5	0.48	0.44	0.38	0.48	0.29	0.46	0.27	0.76	0.47	0.59	0.31
M0t_3515	14.12	14.99	15.38	19.51	18.07	16.21	19.29	17.48	20.73	18.75	18.71	18.09	3.33	2.73	2.95	3.52	2.8	4.43	3.12	3.97	3.92	4.78	5.01	4.72
M0t_14875	12.15	10.8	14.15	23.46	18.96	20.75	20.79	21.31	21.05	8.58	11.17	8.75	5.64	9.15	7.05	8.05	8.03	8.99	6.44	9.44	7	9.24	9.05	6.43
M0t_18922	20.23	18.85	22.99	16.48	19.11	17.75	18.06	14.88	19.03	23.36	18.12	20.4	32.42	33.19	34.82	30.65	33.21	31.67	34.25	34.39	30.77	33.04	37.12	45.71
M0t_1895	13.63	16.83	16.78	19.75	19.47	16.69	18.32	17.09	16.27	15.83	18.56	20.2	11.69	10.92	11.38	9.71	9.44	9.88	10.92	11.76	9.27	10.74	12.14	11.42
M0t_3859	235	21.48	20.6	22.3	21.65	22.27	19.15	21.25	20.51	22.17	23.19	20.19	11.57	13.42	12.17	12.68	11.14	11.18	12	13.29	14.28	11.82	11.58	11.99
M0t_11186	26.55	28.79	30.78	25.83	21.71	6.28	30.58	26.45	29.02	26.71	76	71	275.42	277.85	266.24	224.93	216.53	220.48	197.66	185.12	176.9	182.69	204.97	192.53
M0t_22110	19.79	25.39	26.05	23.39	23.06	22.55	23.47	26.12	21.65	89	77	41	7.09	6.77	5.46	7.49	5.67	4.72	9.04	5.91	4.84	8.04	7.49	8.08

ID	FPKM																							
	K0-1	K0-2	K0-3	K1-1	K1-2	K1-3	K4-1	K4-2	K4-3	K7-1	K7-2	K7-3	R0-1	R0-2	R0-3	R1-1	R1-2	R1-3	R4-1	R4-2	R4-3	R7-1	R7-2	R7-3
M0t_14648	22.15	23.9	25.21	25.65	25.17	22.38	23.41	21.6	23.99	26.23	25.42	21.72	12.29	11.18	17.61	9.77	11.44	11.78	11.48	10.91	13.33	15.72	14.24	14.11
M0t_18123	23.41	25.58	21.34	27.5	27.46	21.61	25.3	26.34	25.54	24.42	26.01	28.41	11.45	10.9	10.85	14.79	16.47	16.64	16.11	15.53	16.39	12.72	14.16	15.08
M0t_17065	22.71	24.98	25.38	31.01	31.09	31.2	24.08	26.05	22.62	25.04	24.85	21.75	13.57	13.44	10.79	11.65	12.03	10.7	11.77	11.31	10.87	10.69	11.78	12.91
M0t_1138	26.21	26.02	28.08	33.71	32.53	31.21	30.44	33.68	32.31	28.69	31.68	31.39	14.63	13.98	15.8	25.65	23.64	29.12	30.78	30.48	34.31	28.9	32.4	30.97
M0t_14411	22	21.79	22.42	32.68	34.96	34.59	29.52	32.82	29.42	33.74	32.52	33.09	15.78	20.34	17.82	21.07	23.59	23.16	21.03	20.26	23.31	23.39	25.71	17.96
M0t_21321	40.72	38.04	41.64	40.92	38.48	44.98	44.28	36.96	34.23	39.82	48.33	48.03	9.53	8.14	2.95	11.31	15.86	18.46	29.43	18.82	30.54	18.25	18.28	15.04
M0t_13779	21.63	22.09	16.92	38.68	43.69	40.04	30.28	40.27	37.52	40.18	42.82	33.67	0	0	0	0	0	0	0	0	0	0	0	0
M0t_17598	73.92	41.93	44.44	80.12	50.89	74.66	65.7	69.19	51.85	76.8	85.33	95.06	35.96	32.42	34.13	48.48	58.07	49.49	44.67	51.44	44.8	42.87	41.26	46.85
M0t_18803	30.84	33.51	34.51	63.13	60.71	67.02	71.53	71.25	77.84	55.76	69.12	63.19	150.52	160.85	145.81	157.95	150.54	161.12	159.46	150.36	153.85	141.64	163.52	140.02
M0t_19302	58.04	56.1	49.53	76.08	73.99	83.07	91.43	84.39	82.06	77.86	82.61	85.35	24.9	25.42	25.93	30.79	32.14	39.66	28.62	25.82	27.86	34.81	34.97	33.98
M0t_17312	59.81	59.25	57.72	75.36	76.17	72.95	70.14	67.62	69.92	65.09	66.14	67.95	32.71	29.16	28.79	35.62	35.94	37.87	34.53	35.77	34.39	31	32.51	28.04
M0t_13866	83.13	85.21	86.86	114.53	111.85	115.97	104.97	113.48	110.87	98.77	107.95	100.69	59.25	53.93	58.9	48.43	45.48	42.94	47.44	42.18	45.16	61.58	58.72	58.92
M0t_17275	119.64	124.21	116.49	145.91	140.68	136.72	135.82	134.2	138.14	132.37	134.77	129.86	0.13	0.36	0	0	0.06	0	0.27	0	0.19	0.12	0.07	0.08
M0t_14684	139.1	152.53	152.89	121.14	143.45	128.95	159.44	150.42	188.12	137.75	148.96	147.43	83.32	80.32	74.46	72.67	73.21	74.77	67.31	60.02	58.18	73.84	64.71	66.21
M0t_19532	216.52	203.4	195.83	179.6	170.6	188.24	195.73	189.31	197.03	185.74	213.22	220.6	4	4.55	3.36	33	2.87	3.55	2.63	3.57	2.86	3.15	3.2	3.85
M0t_4984	8.18	8.26	8.11	8.23	7.92	6.9	8.82	7.28	7.49	8.44	7.48	7.56	8.97	6.17	7.32	6.05	6.21	5.99	6.75	5.97	6.73	8.51	7.11	8.69
M0t_1554	31.99	30.59	31.19	34.44	29.11	30.37	31.78	30.89	31.96	32.18	30.38	32.64	8.3	7.78	10.11	7.42	6.94	10.21	7.53	8.24	7.11	9.26	9.83	8.61
M0t_1801	45.06	43.22	43.01	49.12	46.86	0.15	4.21	43.86	44.13	47.78	47.28	44.12	R0-1	R0-2	R0-3	R1-1	R1-2	R1-3	R4-1	R4-2	R4-3	R7-1	R7-2	R7-3

Supplementary Table S6. Genebank accessions used in Alignment sequences in figure 5

Species	Genbank number
Citrus sinensis	KAH9758124.1
Glycine max	XP 003524661.1
Triticum aestivum	XP 044402740.1
Manihot esculenta	XP 021601648.1
Gossypium raimondii	XP 012446627.1
Hevea brasiliensis	XP 021678755.2
Populus trichocarpa	XP 024451715.2
Arabidopsis thaliana	CAA90809.1

Supplementary Table S7. Genebank accessions used in phylogenetic tree

Species	Genbank number
Actinidia eriantha	XP 057496013.1
Actinidia rufa	GFY90033.1
Rhododendron vialii	XP 058216115.1
Cornus florida	XP 059652944.1
Malania oleifera	XP 057964171.1
Malus domestica	NP 001315871.1
Arabidopsis thaliana	CAA90809.1
Triticum aestivum	XP 044402740.1
Cinnamomum micranthum f. *kanehirae*	RWR81404.1
Magnolia sinica	XP 058070126.1
Vitis vinifera	RVX23236.1
Glycine max	NP 001238087.1
Senna tora	KAF7809869.1
Camellia lanceoleosa	KAI8015846.1
Camellia sinensis	XP 028080167.1
Camellia japonica	WQQ41737.1
Cucurbita pepo subsp. Pepo	XP 023547044.1
Lupinus angustifolius	XP 019438536.1
Populus trichocarpa	XP 024451715.2
Durio zibethinus	XP 022772895.1
Gossypium raimondii	XP 012446627.1
Ziziphus jujuba	XP 015884347.2
Citrus sinensis	KAH9758124.1
Prunus dulcis	XP 034200303.1
atropha curcas	AIT52202.1
Manihot esculenta	XP 021601648.1
Hevea brasiliensis	XP 021678755.2
*Pistacia ver*a	XP 031272387.1
Mangifera indica	XP 044476069.1

Supplementary Table S8. Primer sequences used in this study

Primer name	Sequence	Function	Amplicon size (bp)	Melting Temperature (℃)	GC %
AaMYB44-F	ATGGAGAACCGATCATTGG	Cloning cDNA full-length	837	53.0	47.37
AaMYB44-R	TCAGTCAATTTTGCTGATTCC	Cloning cDNA full-length	837	51.7	38.1
QAaMYB44-F	CTCAACGGGAGAACGGACAA	qRT-PCR	222	57.4	53.5
QAaMYB44-R	GTTGAGAAGAATGCGCGACC	qRT-PCR	222	57.4	53.3
Actin-F	TGCAGACCGTATGAGCAAAG	qRT-PCR	210	55.9	46.6
Actin-R	CCGTCATGGAAACGATGTCT	qRT-PCR	210	57.7	46.9
AtACT8-F	TGCAGACCGTATGAGCAAAG	qRT-PCR	210	55.4	50
AtACT8-R	CCGTCATGGAAACGATGTCT	qRT-PCR	210	55.4	50
AtCBF1-F	GTCTCAACTTCGCTGACTCGG	qRT-PCR	185	62.7	57.1
AtCBF1-R	CATATAAAACGCACCTTCGCTC	qRT-PCR	185	59.0	45.5
AtCBF3-F	TGAACTCATTTTCTGCTTTTTCTGA	qRT-PCR	200	55.4	32.0
AtCBF3-R	AATCCTTGTTTTCTTGTTTGGTTCT	qRT-PCR	200	55.4	32.0
AaMYB44-18803-F	ACAGCCCAAGCTTGCATGCCTGCAGATGGAGAACCGATCAT	Construction of pMD19-T vector	263	60.1	53.6
AaMYB44-18803-R	CCTCGCCCTTGCTCACCATGGATCCGTCAATTTTGCTGATTC	Construction of pMD19-T vector	263	59.8	52.3
AaMYB44-1842-F	TCGCAAGACCCTTCCTCTAT	PCR identification	211	56.8	50
AaMYB44-1842-R	CGACCATTACTCCTTCGATTC	PCR identification	211	56.8	50
PEGFP-N-3-R	CGTCGCCGTCCAGCTCGACCAG	PCR identification	278	55.4	76
